# Advances in the research of immunomodulatory mechanism of mesenchymal stromal/stem cells on periodontal tissue regeneration

**DOI:** 10.3389/fimmu.2024.1449411

**Published:** 2025-01-03

**Authors:** De-Zhi Zhao, Rui-Lin Yang, Han-Xiao Wei, Kang Yang, Yi-Bing Yang, Nuo-Xin Wang, Qian Zhang, Fang Chen, Tao Zhang

**Affiliations:** ^1^ Key Laboratory of Cell Engineering of Guizhou Province, Affiliated Hospital of Zunyi Medical University, Zunyi, Guizhou, China; ^2^ Department of Human Anatomy, Zunyi Medical University, Zunyi, Guizhou, China; ^3^ Department of Prosthetics, Affiliated Stomatology Hospital of Zunyi Medical University, Zunyi, Guizhou, China

**Keywords:** periodontal tissue regeneration, mesenchymal stem cells, periodontitis, alveolar bone loss, immunomodulation, inflammatory microenvironment

## Abstract

Periodontal disease is a highly prevalent disease worldwide that seriously affects people’s oral health, including gingivitis and periodontitis. Although the current treatment of periodontal disease can achieve good control of inflammation, it is difficult to regenerate the periodontal supporting tissues to achieve a satisfactory therapeutic effect. In recent years, due to the good tissue regeneration ability, the research on Mesenchymal stromal/stem cells (MSCs) and MSC-derived exosomes has been gradually deepened, especially its ability to interact with the microenvironment of the body in the complex immunoregulatory network, which has led to many new perspectives on the therapeutic strategies for many diseases. This paper systematically reviews the immunomodulatory (including bone immunomodulation) properties of MSCs and their role in the periodontal inflammatory microenvironment, summarizes the pathways and mechanisms by which MSCs and MSC-EVs have promoted periodontal regeneration in recent years, lists potential areas for future research, and describes the issues that should be considered in future basic research and the direction of development of “cell-free therapies” for periodontal regeneration.

## 1 Introduction

Periodontal tissue, a supportive structure comprised of various mineralized and non-mineralized tissues, including osteoid on the root surface of the teeth, alveolar bone, periodontal ligament, and gingiva (1). The concept of periodontal tissue regeneration refers to the regeneration of periodontal soft tissue, cementum, and alveolar bone. This process encompasses the emergence of new dentin, the reconstruction of functional periodontium, and the formation of fresh alveolar bone, among which the newborn and repair of alveolar bone is the basis of periodontal tissue regeneration. Periodontitis is a chronic inflammatory disease caused by oral microbial dysbiosis with plaque biofilm as the initiating factor and is one of the periodontal diseases with higher incidence and more severe symptoms. Its clinical features are destruction of periodontal supporting tissues, including gingival recession, attachment loss and alveolar bone resorption. In the clinical management of periodontal disease, periodontal tissue regeneration is usually used as the final treatment goal of periodontitis, under the premise of basic periodontal treatment and good control of inflammation, to realize tissue regeneration and functional reconstruction of periodontal lesions, or to accommodate subsequent restoration and implantation treatments.

Periodontitis is the leading cause of tooth loss in adult population and tooth loss, as a major symptom of advanced periodontitis, is inevitably associated with destruction and resorption of alveolar bone. The extensive loss of alveolar bone also poses great difficulties for subsequent prosthetic restoration or implant placement. Therefore, alveolar bone regeneration is clinically necessary to better restore patients’ masticatory function and oral and maxillofacial morphology (2). Research into the regeneration and reconstruction of periodontal supporting tissues has continued unabated over the past decades, ranging from the study of alveolar bone regeneration mechanisms to the exploration of the feasibility of new biological scaffolds to guide periodontal tissue regeneration, to the field of stem cells, which has attracted great attention in the past decade, the study of regenerative medicine has gradually deepened and become more popular.

Mesenchymal stromal/stem cells (MSCs), adult stem cells with multidirectional differentiation potential, are ubiquitously found in tissues such as bone marrow, adipose, muscle, peripheral blood, umbilical cord, and placenta (3). In recent years, MSCs have emerged as a focal point of research in the realm of tissue repair and regeneration, both in basic and clinical medicine, due to their exceptional accessibility, expansion capabilities, and multidirectional differentiation. Within dentistry, both odontogenic MSCs [e.g. Periodontal ligament stem cells (PDLSCs), Dental follicle stem cells (DFSCs), Dental-pulp-derived stem cells (DPSCs), Gingival mesenchymal stem cells (GMSCs), Stem cells from human exfoliated deciduous teeth (SHED)] and non-odontogenic MSCs [e.g. Bone marrow mesenchymal stem cells (BMSCs), Adipose-derived stem cells (ADSCs)] have been employed as seed cells for periodontal tissue engineering, demonstrating significant effects on periodontal tissue regeneration (**Table 1**). MSCs exhibit a beneficial immunomodulatory effect. This is primarily attributed to their low immunogenicity, characterized by negligible or minimal expression of MHC-II antigens (**Table 2**). Consequently, they cannot activate immune effectors such as lymphocytes, enabling them to be xenotransplanted for tissue repair (21). Furthermore, numerous studies have demonstrated that MSCs can suppress immune cell functions, including the proliferation and differentiation of T and B lymphocytes, as well as the secretion of inflammatory factors.

**Table 1:** Characteristics of common MSCs and their use in periodontal tissue regeneration.

Cell source	Cell type	Origins	Position	Difficulty of obtaining	Characterization	Scale of studies in periodontal tissue regeneration	Reference
odontogenic stem cells	PDLSC	neural crest	periodontal membrane and periodontal tissue	easy	good osteogenic and neurogenic differentiation ability	the most extensive	(4, 5)
	DFSC	neural crest	periodontal tissue	easy	strong plasticity	less, more used for periodontal membrane and cementum regeneration	(6–8)
	DPSC	neural crest	healthy pulp	hard	certain anti-apoptosis and neurotrophic ability	less, more focus on the study of dentin-pulp complex	(7, 9, 10)
	SHED	neural crest	deciduous tooth pulp	easy	outstanding neurogenic differentiation ability	comparatively more	(10, 11)
	GMSC	mesoderm, neural crest	gingival tissue	easy	good immunomodulatory properties	less	(12–15)
Non-odontogenic stem cells	ADSC	mesoderm	adipose tissue	easy	good osteogenic potential, convenient materials, sufficient sources	less	(16)
	BMSC	mesoderm, ectoderm	extensive: hematopoietic system, stromal system	easy	The discovery was earlier, and the research was more mature; Its cell differentiation potential is microenvironment dependent	comparatively more	(17)

**Table 2:** Surface marker molecules of exosomes derived from human mesenchymal stem cells/human mesenchymal stem cells.

	hMSCs	hMSCs-EV
+	Stro-1,HLA-I,CD29,CD44,CD71, CD90, CD73, CD105, CD117, CD124, CD133, CD146, CD166, etc. (18, 19)	Alix,TSG101,CD63,CD9,CD81,HSP70,MHC-I/MHC-II,hsp, etc. (20)
–	HLA-II,CD11b, CD14, CD19, CD31, CD34, CD45, CD56, CD79a, etc. (18, 19)	CD14,CD19,CD31,CD45,etc. (20)

In the treatment of periodontal disease, the complexity of the periodontal tissue structure and its immune microenvironment is thought to be one of the reasons why periodontal tissue regeneration is difficult to achieve (22). Conventional treatments provide limited external control of inflammation and infection through mechanical scaling and do not regenerate lost alveolar bone and connective tissue. In contrast, after stem cell transplantation, a small number of stem cells can differentiate into tissue cells with specific functions in the area of periodontal lesions and directly participate in damage repair or even achieve cell replacement for tissue repair. At the same time, most of the stem cells secrete bioactive substances (e.g. growth factors, cytokines, chemokines) through exocytosis and other mechanisms to improve the regenerative environment of the periodontal lesion area from within the body, thus promoting periodontal tissue repair through the principle of cellular empowerment, and also providing the possibility of periodontal bone regeneration and complete regeneration of connective tissues. This paracrine effect varies according to different immune microenvironments, receptor cells and mechanisms. Extracellular vehicles (EVs) constitute a heterogeneous class of membranous vesicles that are incapable of replication. These vehicles exhibit diverse forms, perform a variety of functions, and mirror the physiological state of their cellular origin. They can/secret and produced by nearly all living cells. Depending on their cellular biogenesis, EVs can be broadly classified into three categories: exosomes, micro-vesicles, and apoptotic vesicles. The latest guidelines from the International Society for Extracellular Vesicles (ISEV) (2023) recommend the use of the generic term “EV” and its operational extensions (23). This is to avoid terms with inconsistent and sometimes misleading definitions, such as “exosome” or “micro-vesicle”, which are associated with difficult-to-establish biogenesis pathways. Therefore, it should be noted that in this paper, “MSCs-EV” refers specifically to exosomes of MSCs origin.

The periodontal immune microenvironment is a complex regulatory system composed of various host immune cells, extracellular matrix, and cytokines. These components regulate and influence each other, and any blockage or hyperfunction of any one of them may disrupt the overall regulation of the periodontal environment. Consequently, it is imperative to investigate the host immune regulation and the temporal dynamics of the periodontal immune microenvironment in the context of periodontal disease. Recent studies have elucidated that MSCs engage in intimate interactions with various immune and stromal cells at periodontal lesion sites. These interactions dictate the trajectory of disease progression and influence tissue regeneration. Concurrently, numerous researchers have investigated effective strategies and therapies that harness MSCs and their bioactive cargo to modulate host immune responses (24). This exploration is grounded in periodontal histopathology and immunology, offering a continuous stream of novel targets and insights for immunological studies and therapeutic approaches in periodontal diseases. In this review, we provide a comprehensive overview of MSC properties, advancements in research on immunoregulatory mechanisms during periodontal tissue regeneration and delineate the roles of MSCs in the interplay between the periodontal immune microenvironment, periodontal disease, and bone regeneration within the realms of periodontology and immunology/bone immunology. We also address current challenges faced by MSCs and MSC-derived extracellular vehicles (MSC-EV) in scientific research and discuss their implications for future directions in immunotherapy for periodontal diseases.

## 2 Important factors and pathways associated with the development and regression of periodontal inflammation

Localized plaque microorganisms primarily drive periodontal inflammation and bone destruction. These factors trigger an inflammatory response and bone resorption, which involve the release of endogenous cytokines from various immune cells and osteoclasts (OC). Some of these inflammatory factors act on different cells, mediating processes such as the inflammatory response and degradation of the bone matrix. They also contribute to secondary damage to periodontal tissues. Additionally, certain growth factors have a chemotactic effect on periodontal tissue regeneration. This is achieved by enhancing the ability of periodontal membrane cells to synthesize proteins, thereby contributing to the repair of periodontal tissues. In terms of periodontal bone regeneration, the recruitment of osteogenic precursor cells in the defect area and the induction of osteoblast (OB) formation are closely related to various regulatory factors and targeting pathways.

### 2.1 transforming growth factor-β

TGF-β, a pluripotent cytokine, exhibits direct cytostatic and anti-inflammatory effects. It exists in three isoforms: TGF-β1, TGF-β2, and TGF-β3. TGF-β3 facilitates the homing of endogenous MSCs and enhances tissue regeneration *in situ* by upregulating the secretion of monocyte chemoattractant protein-1 (MCP1) from vascular cells. This process primarily occurs through an indirect, smad3-dependent mechanism that accelerates MSC recruitment and initiates bone formation (24). In addition to its role in cartilage and odontoblast development, TGF-β primarily regulates bone repair in humans, upregulating genes associated with osteogenesis to maintain the balance between bone resorption and formation (25). Its role in inhibiting local inflammation during periodontal bone regeneration has also been increasingly recognized (26). A 2020 study found that varying levels of TGF-β1 had contrasting effects on osteogenic differentiation and bone healing. The mechanism may involve low-dose TGF-β1 activating smad3 to bind to the Bmp2 promoter, thereby upregulating Bmp2 expression in BMSCs. Conversely, high-dose TGF-β1 inhibited BMSC osteogenesis and diminished bone regeneration *in vivo* (27).

### 2.2 Matrix metalloproteinases

Matrix metalloproteinases (MMPs) are secreted by neutrophils, activated macrophages, and epithelial cells in periodontal inflammatory tissues. These include collagenases, elastases, and acid proteases, which play a crucial role in the destruction of periodontal tissue. The concentration of MMPs significantly increases during the active stage of periodontitis, making them an objective test indicator for determining whether periodontitis is active or inactive (28).

### 2.3 Interleukin family

The primary isoforms of IL-1 encompass IL-1α, IL-1β, IL-1SS and IL-1 receptor antagonist (IL-1Ra), among others. IL-1 is a quintessential periodontal proinflammatory factor (29). In the context of periodontitis, IL-1 stimulates osteoclast activity, enhances the production of metalloproteinases, and intensifies collagen degradation within the periodontal matrix. Notably, IL-1β is deemed one of the principal destructive agents in periodontal tissues, making its concentration measurement a valuable diagnostic indicator for active periodontitis. IL-2 is predominantly secreted by Th1 cells, and recent research posits that genetic polymorphisms in IL-2 may be linked to the pathogenesis and prevention of periodontitis (30). IL-4 prompts the production of periodontal anti-inflammatory factors, such as CCL11 (31), and suppresses the transcription of proinflammatory factors, thereby impeding the progression of periodontitis. Furthermore, it has been postulated that IL-4 inhibits alveolar bone resorption by curtailing the formation of osteoclastogenic mediators via the IL-4/CCL22/CCR4 axis (32). IL-6 is a multifunctional cytokine that can be secreted by adipocytes and immune cells. The prevailing view is that IL-6 plays a pivotal role in exacerbating periodontal inflammation and facilitating osteoclastic bone resorption. However, recent studies have highlighted that IL-6 might exhibit an opposing effect in specific environments, such as inhibiting bone resorption and promoting the osteogenic differentiation of hPDLSC (33). IL-8 is a chemokine with pronounced effects on neutrophils, macrophages during inflammation, lymphocytes, and more.IL-10, an anti-inflammatory factor primarily derived from T cells and their subpopulations, plays a crucial role in the development of periodontitis. It inhibits this disease by acting on various immune cells. IL-13, a multifunctional helper T-cell 2 (Th2) cytokine, mitigates inflammatory responses. It can impede the progression of periodontitis by inducing the upregulation of TGF-β and downregulating MMP-1 production, both of which are involved in the regulation of collagen homeostasis in gingival fibroblasts (34). IL-17, another multifunctional cytokine mainly secreted by Th17 in periodontal tissues, contributes to the development of inflammation and bone destruction in periodontitis. In the mouse model of experimental periodontitis, it has been observed that the IL-17/Th17 response leads to the exacerbation of bone loss in early-stage periodontitis. However, IL-17 exerts some osteoprotective effects in advanced periodontitis by regulating the RANKL/OPG ratio (35). IL-23 also influences the development of periodontitis. Numerous studies have shown that elevated levels of IL-23 are directly correlated with the destruction of periodontal supporting tissues.

### 2.4 TNF-α and IFN-γ

Tumor necrosis factor-α (TNF-α) is a pro-inflammatory cytokine that not only promotes the secretion of PGE2 but also stimulates the maturation of pro-osteoclasts and enhances bone resorption. Several studies have demonstrated that inhibiting TNF-α can effectively impede osteoclast formation. Interferon-γ (IFN-γ), another pro-inflammatory cytokine primarily secreted by Th cells, exhibits a dual function in tissue regeneration. On one hand, it encourages collagenase synthesis, while on the other, it restrains the proliferation of fibroblasts. These effects are contingent upon the specific injured tissues and the prevailing microenvironmental conditions. It has been observed that IFN-γ augments osteoclast genesis and periodontal destruction, leading to periodontal bone loss (36). Additionally, IFN-γ suppresses the activity of exogenous mesenchymal stem cells, which are crucial for bone regeneration. Conversely, a study by Gao Y et al. identified two opposing properties of IFN-γ: it directly inhibits osteoclast formation by targeting osteoclast precursors and indirectly promotes osteoclast genesis by stimulating antigen-dependent T-cell activation, causing these T-cells to secrete osteoclastogenic factors such as RANKL and TNF-α. However, during systemic inflammation, these opposing forces achieve a net balance that favors bone resorption (37).

### 2.5 Bone morphogenetic proteins

Bone Morphogenetic Proteins (BMPs) are among the most potent growth factors contributing to bone formation. Except for BMP-1, all members of the BMP family belong to the TGF-β superfamily. These proteins play a crucial role in the development and reconstruction of maxillofacial and alveolar bone (38). They also promote the proliferation of human periodontal ligament cells (hPDLCs) and facilitate the mitotic division of undifferentiated mesenchymal stromal cells in periodontal tissues, leading to their differentiation into osteoblasts and odontogenic osteoblasts. hPDLCs possess properties like those of MSCs, making them suitable for xenografts. Furthermore, they contribute to the regulation of microenvironmental homeostasis and tissue regeneration in periodontal connective tissues.

### 2.6 OPG/RANK/RANKL

Osteoprotegerin (OPG) inhibits the formation of osteoclasts, and an increase in OPG secretion leads to increased bone mass. Conversely, osteoporosis is observed in mice deficient in OPG. The receptor activator of NF-κB (RANK) serves as the sole target signaling receptor on the surface of osteoclast precursor cells. It activates these cells by binding to RANKL, thereby initiating a signaling program. The ligand for the nuclear factor kB receptor activator of NF-κB (RANKL) is expressed on the surface of osteoblasts and stimulates the differentiation and maturation of osteoclasts. OPG can function as a decoy receptor for RANKL, competing with or antagonizing RANK (42). This prevents the activation of the RANKL/RANK pathway during the osteoclast process, thereby inhibiting bone resorption. Both OPG and RANKL are expressed in periodontal tissues. An increase in RANKL secretion leads to hyper resorption of periodontal bone. The OPG/RANK/RANKL triad is central to the bone immunity/bone regeneration regulatory pathway. Other periodontal cytokines and hormones lack corresponding receptors on the osteoclast surface, resulting in minimal modulation of bone regeneration by other pathways. The balance between MSCs (MSC-EV) and the OPG/RANK/RANKL signaling axis has been shown to be critical for the success of periodontal bone regeneration, orthodontic treatment and implant restoration (43, 44).

### 2.7 Wnt pathway

The WNT family comprises a group of secreted glycoproteins that influence neural crest-derived cells via various signaling pathways. These pathways regulate the development of teeth and maxillofacial bone, as well as participate in the regulation of bone homeostasis and density during maxillofacial bone remodeling. The signal transduction pathways can be broadly categorized into the Wnt classical pathway and the Wnt non-classical pathway, also known as the Wnt/β-Catenin pathway. This pathway is activated by Wnt1, Wnt3a, Wnt8, etc., and stimulates target genes by stabilizing β-Catenin in the nucleus. The non-classical Wnt pathway, activated by Wnt4, Wnt5a, etc., can function at both transcriptional and non-transcriptional levels. In mouse models, osteoblasts can highly express WNT proteins. After co-culturing with MSCs, the Wnt signaling pathway in osteoblasts is activated, leading to enhanced osteogenic properties. It has been demonstrated that Wnt5a regulates RANKL expression in periodontal ligament cells (PDLC) (45).

The Wnt signaling pathway has been a major focus in recent years in the study of oral and maxillofacial tissue regeneration and tissue engineering. Recent studies in the last two years have shown that hPDLSCs regulate periodontal tissue homeostasis and periodontal regeneration through different Wnt pathways, and that classical and non-classical Wnt pathways are triggered by different mechanisms (33). And activation of the Wnt/β-catenin pathway can regulate the phenotypic switch of macrophages in periodontal tissues (promoting polarization from M1 to M2 phenotype) (46), which plays a role in suppressing immune dysregulation and promoting periodontal regeneration.

## 3 Regulation of immune cells by MSCs in periodontal tissue regeneration

The body’s immune system maintains physiological homeostasis and defends against pathogens by inducing immune responses, as is the case within the periodontal immune system. Immunomodulation is the role of the body’s immune system in controlling the type and intensity of the immune response and is primarily immunosuppressive in nature. The basic cells involved in immunomodulation are the immune cells, and these different types of regulatory cells interact in a variety of ways with some of the immunomodulatory molecules to keep the body’s immune response within homeostatic limits. Immune cells are broadly divided into lymphocytes and myeloid cells: lymphocytes include T cells, B cells and natural killer (NK) cells; myeloid cells are derived from myeloid precursor cells in the bone marrow, including neutrophils, monocytes/macrophages, mast cells, dendritic cells, etc. In the following, we state the relationship between the immunomodulation and action of MSCs on different types of immune cells.

### 3.1 T lymphocytes and their subpopulations

**Figure 1:**
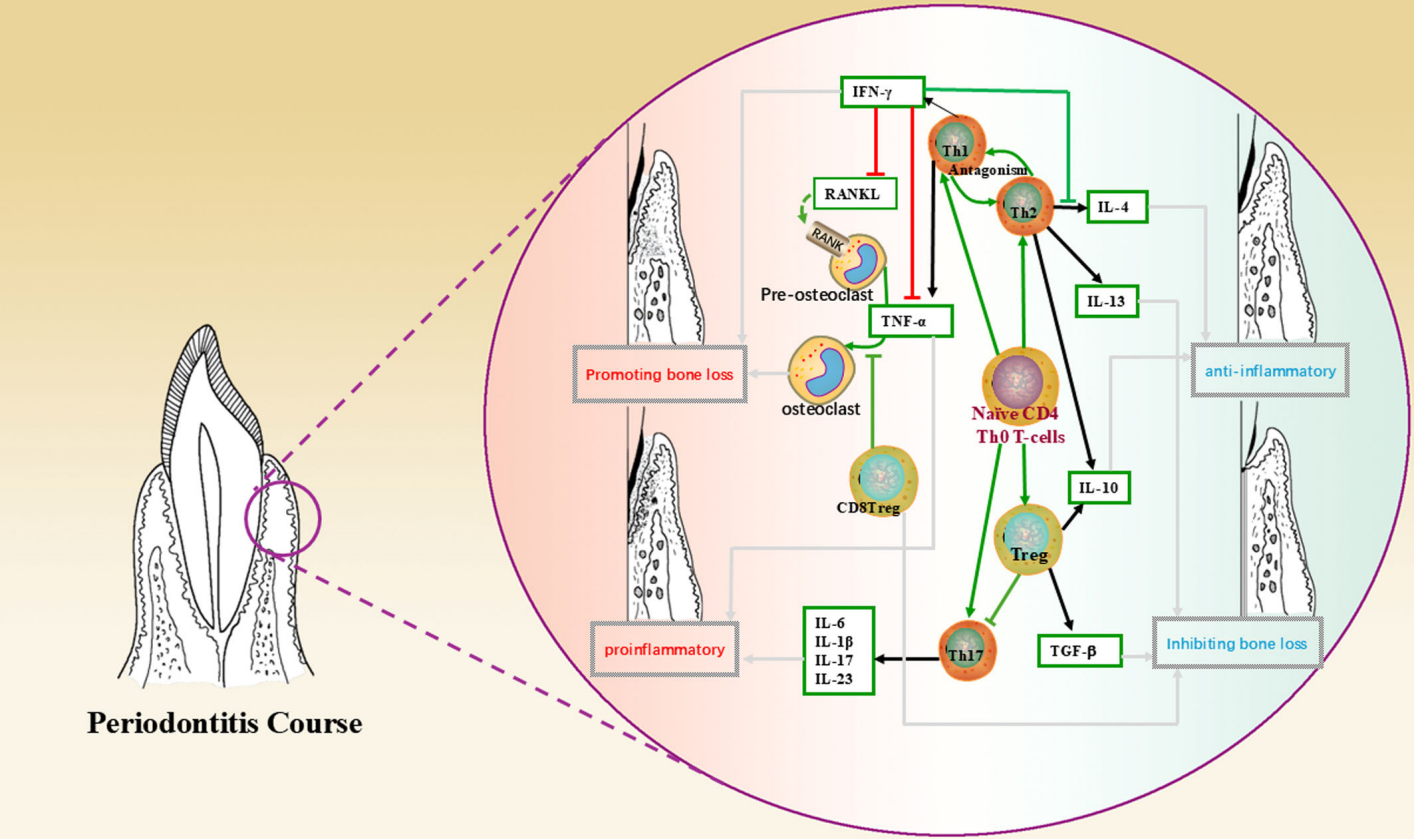
This figure shows the mechanism of action of T lymphocytes and their subpopulations, cytokines in the periodontal microenvironment and the direction leading to disease regression, where green arrows indicate the direction of cell differentiation; black arrows indicate secretion, production of cytokines; red lines indicate promotion, up-regulation; green lines indicate inhibition, down-regulation; and grey arrows indicate the direction of bringing about a reversion in the nature of the microenvironment.

The immunomodulatory effects of MSCs on T cells are particularly complex (47). It can be achieved through direct contact with immune cells and modulation of the secretion of cytokines, chemokines and growth factors (48). For example, the mechanism of immunosuppression by MSCs through direct contact with lymphocytes is to promote apoptosis through the classical Fas/FasL pathway, which is highly expressed on the surface of lymphocytes and can combine with FasL on the surface of MSCs to initiate the apoptotic program during direct contact (49); it has been found that MSCs inhibit the production of cytokines by T cells (IL-2, TNF-α, IFN-γ, IL-6, etc.) and increase the expression of IL-10 and TGF-β in T cells (50). In addition, MSCs inhibit the proliferation of cytotoxic T cells (CTL), thus inhibiting the production of cytotoxins and playing an indirect role in suppressing the immune response (51). The interleukin family, interferon and some other cytokines secreted by T lymphocytes and their subpopulations play a role in periodontal lesions through different mechanisms and pathways. The following figure briefly describes the network of their roles with cytokines in the development and regression of periodontitis and the involvement of MSCs in the regulation of the same, using T lymphocytes and their subpopulations as an example (**Figure 1**, **Table 3**).

**Table 3:** Mechanism of action of MSCs on T cells in periodontitis/periodontal regeneration.

Important role of MSCs for T cells	Mechanisms	Directions affecting periodontal tissue regeneration	References
Reduced Th17/Treg ratio	EV-miRNA/FOXP3	Enhance the secretion of local anti-inflammatory factors to relieve periodontal inflammation	(52)
Promote differentiation of Th0 cells to Treg cells	HIF-1α/STAT3	Inhibition of periodontal bone loss	(53)
Reduced Th1/Th2 ratio	Secretion of cytokines	Relief of localized periodontal inflammation	(54)
Inhibition of CTL proliferation	–	Inhibits cytotoxic production and thus indirectly suppresses the immune response	(51)
Direct contact with T cells within the microenvironment	Fas/FasL pathway	Directly causes T-cell apoptosis	(49)
Inhibition of inflammatory cytokine production by T cells and increased expression of anti-inflammatory factors in T cells	paracrine secretion	Anti-inflammatory immunosuppressive effects	(50)
Indirect inhibition of T cell proliferation	Secretion of anti-inflammatory cytokines to impair antigen presentation by DCs	Preventing excessive periodontal immune response	(55)

#### 3.1.1 CD4^+^/CD8^+^ T cells

The CD4^+^/CD8^+^ phenotype is a co-receptor for T cells expressing the TCR complex, which is restricted by MHC class II/I molecules on the surface of antigen presenting cells and is therefore differentiated during antigen recognition. CD4^+^ T cells can differentiate into specialized effector subsets that can play a role both directly in protective immunity and in coordinating other immune molecules to play a role in adaptive immunity (56). CD8^+^ T cells express CD8 molecules. Their specialized subsets, CD8^+^ Treg (Except here is special labelling, all other “Treg” in the article refer to CD4^+^ Treg.) have been shown to influence the dynamic balance between osteogenesis and osteoblast genesis in the progression of periodontitis by inhibiting osteoclast genesis and modulating the immune response in the periodontal microenvironment (57). Many studies have found that the ratio of localized periodontal CD4^+^ to CD8^+^ T cells is significantly lower in periodontal inflammation compared to healthy individuals (58). One study found that human periodontal ligament stem cells (hPDLSC) inhibited the proliferation of CD4^+^ T lymphocytes, and their effect was enhanced by IFN-γ and IL-1β (59). In a study of periodontitis in patients with systemic lupus erythematosus (SLE), researchers found a negative correlation between the CD4/CD8 ratio and the periodontal index due to T lymphocyte activation in SLE, suggesting that the ratio may be a measure of the severity of periodontitis in SLE patients (60).

It is worth noting that in recent years, regulatory T cells (Treg) and helper T cells (Th cells) have received increased attention, such as the migration and recruitment of Treg cells in the microenvironment, the cooperation between Treg cells and other types of immune cells, and the distinct roles played by Treg cells in different disease processes, etc. (60–64). Both Treg and Th cells belong to the subpopulation of CD4^+^ T cells. Th cells include T helper 1 (Th1), T helper 2 (Th2), T helper 17 (Th17), T helper 9 (Th9), etc. These cells need different cytokines. These cells require different cytokines and transcription factors to be activated and are also capable of secreting different cytokines that have different effects in different immune microenvironments. For example, Th1 cells secrete IFN-γ and TNF-α to exert pro-inflammatory and pro-bone loss effects in the local microenvironment, whereas Th2 cells mainly secrete anti-inflammatory factors such as IL-10 and IL-4 to counterbalance Th1 function. MSCs induce Th1/Th2 in antagonistic relationships towards Th2 cells for anti-inflammatory immunomodulatory effects (54). The relationship between Treg and Th17 cells is highlighted below.

#### 3.1.2 Treg/Th17 cells

Treg are a naturally occurring subset of T cells in the normal human immune system with immunosuppressive effects, characterized by high expression of the transcription factor Foxp3. Upon antigenic stimulation, Treg cells can be differentiated from peripheral mature CD4^+^ T cells into inducible Treg (iTreg/adaptive Treg, aTreg), which regulate the adaptive immune response. Treg cells regulate the level of inflammation and maintain immune homeostasis by secreting IL-10, IL-12 and transforming growth factor-β. Th17 cells are differentiated from Th0 cells by antigen presentation or stimulation with pro-inflammatory cytokines (e.g. IL-1β, IL-6, IL-23). They have been shown to play a role both in host defense against pathogens and in the development of autoimmune diseases, as exemplified by their ability to induce tissue inflammation and secretion of a variety of cytokines (IL-17, IL-6, IL-21, TNF-α, etc.) and to enhance the antimicrobial capacity of neutrophils through the release of factors such as IL-1, IL-8, IL-23R and MMPs.

T lymphocytes in the periodontal microenvironment are similarly differentiated into different populations, including Treg cells and Th17 cells (65). Periodontitis has been shown to disrupt immune homeostasis by enhancing Th17 function and impairing Treg function (66). During the course of periodontitis, Treg and Th17 can also balance the ratio between the two, regulating each other and influencing periodontal bone loss by upregulating/downregulating the inflammatory response (67–69). When Th17 cells are increased and Treg cells are decreased, there is an increase in inflammatory cytokines and alveolar bone resorption, suggesting that Treg cells have a function in inhibiting periodontal bone loss while Th17 cells upregulate the inflammatory response and promote periodontal bone destruction (32). It has been found that local enrichment of Tregs restores local immune homeostasis, prevents bone loss and ameliorates local inflammation in a mouse model of periodontitis (70). Scholars have also found that Treg cells in mice with periodontitis exhibit characteristics of Th17 cells in the mid-stage of the disease, suggesting that Treg cells can transform into Th17 cells and suggesting a high degree of plasticity of Treg cells (71). In addition, many researchers have focused on the impact of the Treg/Th17 balance in innate and adaptive immunity in periodontal disease, for example, it has been proposed that in the presence of immunomodulatory factors such as NLRP3 (a protein complex involved in the regulation of innate immune responses), Foxp3 expression is suppressed, which disturbs the Treg/Th17 balance (i.e., Th17 cell/Treg ratio decreases) and increases the release of inflammatory cytokines, exacerbating periapical inflammation (72) and leading to periodontal damage. Therefore, one possible mechanism is that in early or progressive periodontitis lesions, Treg cells may be compensated to augment and attenuate the adaptive immune response. However, as periodontitis progresses, the inflammation becomes chronic and Treg in the inflammatory microenvironment gradually lose their immunosuppressive function and are gradually dominated by other pro-inflammatory factors and replaced by dominant cells such as Th17.

On the one hand, MSCs can promote the differentiation of Th0 cells towards Treg cells (53), promote the secretion of anti-inflammatory factors, and then inhibit the proliferation of CD4^+^ and CD8^+^ cells, indirectly inhibiting the function of CD4^+^ and CD8^+^ T cells; On the other hand, in the case of inflammatory diseases in the body, the balance of Th cell subsets is disturbed, and MSCs can be used as a regulator to restore or reverse the imbalanced cell populations, such as MSCs can prevent Treg to Th17 conversion and inhibit Th17 function, thus realizing the regulatory effect of Treg/Th17 in periodontal diseases. For example, Zheng Y et al. found that pro-inflammatory PDLSCs-EV could regulate the balance of Th17/Treg cell ratio in the microenvironment of chronic periodontitis and attenuate the inflammatory response (73); A study in 2023 investigated the effect of hBMSC-EVs on the dynamic homeostasis of T cells in periodontitis (66). hBMSC were isolated from the maxilla of healthy volunteers, and by using miRNA sequencing, the differentially expressed miRNAs and target genes in EVs generated from hBMSC stimulated with LPS were investigated, It was found that EV-miR-1246 efficiently reduced the Th17/Treg ratio *in vitro* (resulting in a 75% reduction in the number of Th17 cells measured experimentally), and EV-miR-1246 was shown to promote periodontal regeneration in a mouse model of experimental periodontitis in animal studies. This undoubtedly represents a promising therapeutic target for the targeted treatment of periodontitis.

### 3.2 B lymphocytes and their subpopulations

The main function of B lymphocytes is to mediate humoral immunity. When stimulated by antigens, B cells activate, differentiate into plasma cells and produce antibodies. In addition, B cells and their subpopulations also play a role in immune regulation and tolerance. B cells have many subpopulations, such as B1 cells, B2 cells, B effector cells, and regulatory B cells (Breg).

B1 and B2 cells are classical B lymphocytes involved in intrinsic and humoral immune responses, respectively. While effector B cell subpopulations mainly play a secretory role in a specific immune microenvironment stimulated by specific cytokines, Breg cells, similar to Treg cells, are a type of B cell with a negative regulatory role that can produce large amounts of IL-10 and regulate the balance between Th1 and Th2, thereby suppressing harmful immune responses (74). Numerous studies have demonstrated the immunosuppressive effects of Breg, e.g. Breg can inhibit Th1 and Th17 responses and induce FoxP3+ Treg pools, which play a key role in maintaining peripheral tolerance (69, 75). These cells may become new targets for the treatment of inflammatory diseases and are a hot spot for future research.

A study in periodontal disease confirmed that Breg inhibits inflammation produced by immune cells such as T cells and reduces the expression of pro-inflammatory cytokines through the secretion of IL-10 (B10 cells), and that it inhibits RANKL-expressing neutrophils and blocks alveolar bone loss in experimental periodontitis in mice (76). Thus, Breg plays a role in inhibiting bone loss in periodontitis.

The vast majority of researchers believe that MSCs can inhibit the proliferation and differentiation of B cells through soluble cytokines (TGF-β, PGE2, etc.) and regulate the production and secretory function of Bregs through intercellular contacts, extracellular vesicles and other modalities, and exert an anti-inflammatory effect by promoting the increase of Bregs (69). In a minipig periodontitis model, MSCs block B cells in the G0/G1 phase of the cell cycle through intercellular contacts, inhibit B cell activation and proliferation, and induce Bregs production, thereby reducing humoral immunity, a process mediated mainly through programmed cell death protein 1 (PD-1) and PD-L1 (77). In contrast, MSCs-EV inhibit B cell proliferation and BCR-mediated Ca^2+^ mobilization and regulate the PI3K-AKT signaling pathway, which is an important link in the regulation of Breg cell development and an upstream pathway that regulates macrophage M1/M2 polarization (78). In addition, in contrast to reducing humoral immunity, a previously unrecognized function of PDLSCs in regulating humoral immunity has been proposed, i.e. when co-cultured with normal B cells, PDLSCs enhance B cell activity by upregulating IL-6 secretion (79) and promote B cell proliferation. Previous studies have shown that in active periodontitis pathology, IL-6 can be secreted by the body’s memory B cells, promoting inflammatory infiltration and facilitating periodontal tissue destruction (80). However, it has also been pointed out that IL-6 can also play a role in inhibiting bone resorption when the level of RANKL in the local microenvironment is altered (81), so we believe that in the disease process intervened by MSCs, their effect on the function of B cells at different stages of periodontitis may also vary depending on the developmental stage of the B cells and the changes in the microenvironment (the function of bioactive factors), and the mechanism needs to be further explored.

### 3.3 Monocytes/macrophages

Monocytes are derived from hematopoietic stem cell precursors in the bone marrow and, when stimulated by inflammatory signals, can rapidly reach the site of infection, divide and differentiate into macrophages and dendritic cells. Macrophages can phagocytose pathogenic microorganisms, recognize antigens and relate signaling molecules, secrete inflammatory mediators and initiate intrinsic immune responses.

Macrophages are considered to be a highly heterogeneous population of cells, and the functions of individual populations can differ significantly *in vivo* (82). Among them, M1 (pro-inflammatory phenotype) and M2 (anti-inflammatory phenotype) are functionally distinct and easier to distinguish and are the two phenotypes that play important roles in the inflammatory microenvironment. The mechanism by which stem cells induce macrophage polarization towards the M1/M2 phenotype has been a hot research topic in recent years. For example, macrophage involvement in the development of periodontitis has been well documented (83, 84) and macrophage conversion from M1 to M2 promotes periodontal tissue regeneration and inhibits alveolar bone loss (85). For example, in the early stages of inflammation, such as the development of gingival inflammation and the appearance of periodontal pockets, M1 macrophages predominate and produce pro-inflammatory factors to enhance the inflammatory response. As the inflammation progresses to a certain level, M2 macrophages are gradually activated and play a role by releasing large amounts of IL-10, anti-inflammatory cytokines, to promote periodontal tissue regeneration.

Many studies have demonstrated the role of MSCs in stimulating monocytes/macrophages to convert to the M2 phenotype in the treatment of inflammatory diseases such as periodontitis. In the experiments of Liu J et al (85), the levels of IL-10 were increased in the rat group treated with PDLSCs, while the levels of TNF-α were decreased, suggesting that PDLSCs may promote the repair of periodontal function by influencing macrophage polarization, reducing periodontal tissue inflammation and promoting periodontal tissue regeneration. Gao X et al. (86) First, SHED and monocytes/macrophages were co-cultured in the Transwell system, and then a rat periodontitis model was used to observe the effect of SHED on periodontal regeneration. The result suggests that SHED may promote periodontal bone regeneration by promoting macrophage polarization to the CD206^+^ M2-like phenotype. Pang QM et al. demonstrated that peripheral blood mesenchymal stromal/stem cells (PBMSC) and their culture medium can mediate M2 macrophage polarization through activation of the IL10/STAT3 pathway, which promotes tissue repair and favors functional improvement in inflammatory diseases (87). In addition, a study in 2022 (88) showed that PDLSCs conditioned medium (PDLSCs-CM) significantly increased the expression of anti-inflammatory factors such as IL-10, TGF-β and CCL18, which in turn induced and enhanced IL-4 and IL-13-induced polarization of M2 macrophages.

Furthermore, MSCs can act synergistically with macrophages to enhance bone healing by modulating inflammation and promoting regeneration. For example, MSCs affect macrophage polarization, which in turn affects osteoclast differentiation and bone repair processes. Macrophages can activate endogenous MSCs and promote their differentiation into the osteogenic lineage, which is essential for effective bone tissue engineering and repair (89); macrophages can influence MSC differentiation by releasing extracellular vesicles, such as apoptotic vesicles enriched in miR155 (90). These vesicles regulate MSC fate mainly by modulating pathways involved in osteogenesis and adipogenesis. However, a number of scholars have similarly noted that under specific conditions, the function of MSCs is reversed by the influence of macrophages of different phenotypes, and this process is regulated by the concentration of inflammatory factors, e.g., when there is a lack of IL-6 in the local microenvironment, MSCs induce the differentiation of M0 into M1, which secretes TNF-α and IFN-γ and expresses the co-stimulatory molecule CD86, which contributes to T cell activation, which in turn produces a large number of inflammatory factors and stimulates MSCs to exert anti-inflammatory effects through a negative feedback mechanism. Therefore, the dynamic change of inflammatory factor concentration in the local microenvironment plays an important role in the pro-inflammatory or anti-inflammatory effects of MSCs and the transformation of macrophages of M1 and M2 phenotypes.

Recent studies have shown that macrophages exhibit significant plasticity, and dynamic changes in the local microenvironment can modulate their phenotype to produce changes beyond M1 and M2, as evidenced by different gene expression profiles associated with the M1 and M2 states, as well as intermediate and mixed states that do not fully conform to the M1/M2 dichotomy (91). Studies using advanced technologies such as single-cell RNA sequencing have identified new macrophage subpopulations in the tumor microenvironment, characterized by unique transcriptomic profiles distinct from traditional M1/M2 markers (92). Specific tumor macrophage (TAM) subpopulations have been identified as TREM2^+^, SPP1^+^, etc., each of which has a distinct role in cancer prognosis and treatment response.

Therefore, we believe that the M1 and M2 phenotypes are more inclined to be two idealized, borderline opposing forms of macrophages, and that the M1/M2 classification provides a practical framework for our understanding of macrophage function, but the complexity of macrophage biology suggests that macrophage activation states are continuous and dynamically regulated by the local microenvironment. Therefore, understanding the full spectrum of macrophage activation states can lead to more precise therapeutic strategies, and the interactions between MSCs and different phenotypes of macrophages in the microenvironment will be more precise if a more nuanced approach is adopted in research and therapy; therefore, identifying and targeting specific macrophage phenotypes in disease-targeted therapies is expected to improve the prognosis of a variety of diseases, and further probing into the interaction with the MSCs action of crosstalk will be of great benefit.

### 3.4 Dendritic cells and NK cells

Dendritic cells (DCs) are antigen-presenting cells that capture, process and present antigens to lymphocytes to initiate and regulate adaptive immune responses and are therefore closely related to T lymphocytes with which they can form a unique immunoregulatory network. Dendritic cells direct the differentiation of CD4 T cells into T cell subpopulations such as Th1, Th2, Th17 and Treg cells and maintain their numbers (93). Prolonged exposure of periodontal tissues to oral bacteria stimulates an inflammatory response that leads to gingivitis or periodontitis. Dendritic cells play a destructive role in periodontal tissues by secreting inflammatory factors, mainly through activation of the acquired immune response (94).

The effects of MSCs on periodontal DCs can be direct or indirect. For example, MSCs can come into direct contact with DCs and inhibit their maturation, causing them to remain at a low differentiation stage and thus lose their ability to present antigens to down-regulate periodontal inflammation (95); Monica Reis, et al. confirmed that MSC-EV treatment inhibited DC maturation, reduced the secretion of pro-inflammatory cytokines (e. g., IL-6) in the microenvironment, and increased the secretion of anti-inflammatory cytokines (e. g., TGF- β) (55); MSCs can also stimulate the development of monocytes into DCs with immune-tolerant properties, resulting in an increase in the secretion of IL-10 and a decrease in the secretion of IFN-γ, TNF-α and IL-12, which impairs the antigen-presenting ability of DCs and thus inhibits T-cell activation and proliferation (96). Therefore, this process may play a role in preventing overactivation of the periodontal immune response.

Natural killer (NK) cells are important immune cells in the body that have an immunosurveillance role and can non-specifically kill a variety of target cells without antigenic stimulation and play an important role in regulating the balance of periodontal osteoblasts/osteoclasts (97). MSCs can inhibit the proliferation of NK cells and their secretion of inflammatory factors and reduce their non-specific killing effect on target cells. In recent years, several studies on natural killer T cells (NKT) have reported their immunomodulatory role in periodontal disease. Natural killer T cells are essentially a distinct subpopulation of T lymphocytes with some of the properties of NK cells that produce anti-inflammatory and pro-inflammatory factors involved in immunomodulation and play a predominantly pro-inflammatory role in the development of periodontitis. For example, a recent study showed that in the early stages of alveolar bone injury, NKT cells can interfere with the osteogenic differentiation of MSCs by upregulating inflammatory factors (e.g. CXCL2), thereby adversely affecting alveolar bone repair (98). However, whether MSCs can regulate the function of NKT cells in periodontal regeneration is still unknown and requires further investigation.

### 3.5 Neutrophils

Neutrophils are intrinsic immune cells and important cells in the defense process of periodontitis, with a complex network of interactions with other types of immune cells. In addition to phagocytosis, secretion of antimicrobial molecules and enzymes, neutrophils can synthesize pro-inflammatory mediators to modulate adaptive immunity and activate osteoclasts to promote the development of periodontal inflammation. MSCs can play an immunosuppressive role by inhibiting NO secretion from neutrophils, reducing neutrophil infiltration and other mechanisms (99), and also indirectly modulate the number and activity of neutrophils in the microenvironment and by regulating Breg cells. It may also indirectly regulate the number and activity of neutrophils in the microenvironment by regulating Breg cells.

## 4 MSCs promote periodontal regeneration in the immune microenvironment

### 4.1 MSCs are important regulators of the periodontal immune microenvironment

MSCs play an important immunomodulatory role in periodontal tissue regeneration at different stages of the periodontal disease process. In the early stage of periodontitis, endogenous MSCs can assist the host in activating the immune defense and mediate the expansion of the inflammatory process (100). Meanwhile, as the lesion progresses, MSCs can inhibit the inflammatory response through interactions with immune cells and paracrine mechanisms, causing the periodontal inflammation to pass through the high-level inflammatory stage as soon as possible and preventing the delay and entry into the chronic inflammatory state. For example, gingival tissues, as the most involved site in periodontal inflammation, have native GMSCs that play a pivotal role in regulating inflammation (101). Although GMSCs have been discovered relatively recently, they have been shown to be unique MSCs with inflammation resistance that can maintain stable stemness and multispectral differentiation potential in response to continuous stimulation by inflammatory factors (102). The immunomodulatory function of GMSCs has also been more studied, which is able to inhibit the function of immune cells by synthesizing PGE2 and secreting anti-inflammatory factors such as IDO and IL-10. In addition, under conditions of local inflammation, TNF-α secreted by M1 macrophages in the microenvironment can stimulate GMSCs to produce anti-inflammatory factors such as IL-6, and at the same time increase the number of secretion of GMSCs-EVs and increase the expression level of CD73, which induces the polarization of macrophages toward the M2 phenotype, thus rapidly controlling and reversing the inflammatory environment (45). Recent studies have shown that gingival mesenchymal stem cells derived from rheumatoid arthritis patients(RA-GMSC) can effectively inhibit T-cell proliferation, pro-inflammatory cytokine secretion, and osteoclast differentiation *in vitro*, as well as reduce Th1 and Th17 cell ratios and elevate Treg cell ratios, thus demonstrating significant efficacy in a model of collagen-induced arthritis (CIA) (103). And exogenous transplantation of MSCs may also lead to organic changes in the local microenvironment of tissues, freeing chronic inflammation from the dilemma of stasis and restarting tissue repair of chronic injuries (e.g. spinal cord injury (104) and chronic periodontitis), which may be beneficial for disease treatment., and these processes cannot be separated from the role of MSCs-EVs. And the above regulatory functions are microscopically controlled by the balance and coordination of many signaling pathways and environmental factors.

#### 4.1.1 MSCs-EVs are important players in periodontal immunomodulation

Numerous studies have shown that MSCs enter the organism and their live cell signals are no longer detectable after a relatively short period of time, yet they can have profound and lasting effects on disease treatment and tissue regeneration (105), which brings us to the paracrine role of MSCs.

MSC-EVs are membranous vesicles secreted by living cells from the late nuclear endosome, which is naturally present in body fluids including blood, saliva, urine and breast milk. MSCs can release a variety of biologically active factors through MSCs-EV, such as proteins, lipids, deoxyribonucleic acid (DNA), messenger RNA (mRNA), circular RNA (circRNA), microRNA (miRNA) and other non-coding RNAs (**Table 4**). They act as endogenous carriers of genetic material or chemical mediators, realize information transfer in intercellular communication, regulate the cellular biological activity of recipient cells and also participate in the immune regulation of the organism. For example, HUCSC-EVs have been shown to decrease pro-inflammatory cytokines such as tumor necrosis factor α, IFN-γ and IL-17A, while increasing anti-inflammatory cytokines such as TGF-β and IL-10 in periodontal tissues. This modulation contributes to the attenuation of inflammatory responses associated with periodontitis (120). MSCs pre-treated with TNF-α can produce EVs with enhanced immunomodulatory properties. These EVs inhibit pro-inflammatory markers and enhance repair markers in macrophages, contributing to reduced inflammation and improved bone regeneration in the tissue repair environment (121).

**Table 4:** Content of major cytokines and miRNAs in common MSCs-EV in recent studies.

EV Sources	Cytokine Name	MiRNA Name	Mechanism/Channel	Effect	Reference
PDLSCs-EV		miR-141-3p	KEAP1-NRF2	Alleviation of high glucose-induced senescence and damage and alleviation of oxidative stress in PDLSC	(106)
		miR24-2,miR142,miR335,miR490,miR296	regulate Ras protein signaling	Regulation of proto-oncogenes to maintain normal cellular functions	(107)
		miR143-3p	PI3K/AKT/NF-κB	Promoting M1 macrophage polarization and upregulating local inflammation	(108)
		miR155-5p		Reduce the inflammatory response in chronic periodontitis	(73)
	TNF-α,IFN-γ			Promote localized inflammation	(109)
	VEGF		regulation of angiogenesis and vascular function	Promotion of new blood flow to periodontal tissues	(110)
ADSCs-EV		miR-21-5p,miR-92A	inhibition of PTEN and activation of PI3K/p-Akt	Enhances angiogenesis and promotes adipose tissue regeneration	(111)
		miR-221,miR-222	targeting PUMA and ETS-1	Inhibition of apoptosis and hypertrophy in cardiomyocytes	(112)
	IL-1β, TNF-α,IL-6			Promote localized inflammation	(113)
GMSCs-EV		miR-1260b	Wnt5a/RANKL	Inhibits osteoclast activity and promotes periodontal bone regeneration	(45)
		miRNA-148a	IKKB/NF-κB	modulate Treg/Th17 balance in RA model	(114)
	TGF-β,FGF, VEGF		regulation of cell proliferation and differentiation	Anti-inflammatory, facilitates tissue regeneration and bone repair	(115)
	Wnt family		regulation of cell proliferation and differentiation	Favors tissue development and regeneration	(115)
BMSCs-EV		miR-139-5p	affects HSPC proliferation and apoptosis	Maintenance of hematopoietic dynamic balance	(116)
		miR-140-3p,miR-15b	inhibition of the NF-κB pathway to target osteoblasts	promote osteogenesis	(117)
	IL-6,IL-10,TGF-β,			Anti-inflammatory	(118)
	TNF-α			Promote localized inflammation	(118)
SHED-EV		miR-330-5p	targeting Ehmt2 and mediating CXCL14 transcription	Reducing the inflammatory response to traumatic brain injury	(119)
		miR-100-5p, miR-1246	down-regulation of angiogenesis-related factors such as VEGFA and MMP-9	Inhibition of angiogenesis	(119)
		miR-26A	TGF-β/SMAD2/3	Promoting neovascularization in pulp regeneration	(119)

Consequently, their anti-inflammatory and immunosuppressive effects in different environments can be used as an effective tool in the treatment of periodontitis. Considering that MSCs-EV is an excellent bioactive carrier, there have been numerous studies and explorations of “cell-free therapy” strategies in recent years, and many scientists have attempted to use MSC and MSCs-EV in the treatment of periodontitis and have made preliminary progress in clinical trials (122, 123). However, due to the stability and retention of MSC-EVs *in vivo* after application (124), their main form of application in preclinical studies currently relies on the use of various types of hydrogels, collagen gel sponges, synthetic polymers and other biomaterials to encapsulate them for prolonged tissue retention and maintenance of bioactivity (125).

#### 4.1.2 Bidirectional regulation of MSCs and inflammatory cytokines

The pathology of periodontitis is essentially a tissue defense following bacterial infection, with plaque microorganisms as initiating factors triggering inflammatory responses of varying degrees in the periodontal tissues. The inflammatory microenvironment (126, 127) contains a variety of immune cells and related inflammatory factors that are highly aggregated locally in the inflamed tissues and can recruit and mobilize endogenous MSCs, as well as chemotactic homing of exogenous MSCs, activate the function of MSCs and drive the inflammatory microenvironment in a direction that favors regeneration through immune modulation. Similarly, once the internal and external environment conducive to tissue repair is disrupted, the function of MSCs is inhibited or apoptosis occurs and the local inflammatory cytokine imbalance is restored, which can plunge the tissue back into a prolonged inflammatory environment.

Under normal conditions, the body’s MSCs are controlled and quiescent and do not have the ability to immunomodulate (128). Instead, MSCs can be activated under the stimulation of periodontal inflammatory factors. In a low-inflammatory environment, MSCs can recruit immune cells or promote the formation of immune cells in the microenvironment to promote the development of inflammation, whereas when inflammatory cytokines exceed a certain threshold, MSCs can be converted to an anti-inflammatory phenotype (129), regulating immune cells by releasing anti-inflammatory factors (e.g. IL-4, IL-10) and reducing pro-inflammatory factors (TNF-α, IFN-γ). For example, in an inflammatory microenvironment dominated by IFN-γ and TNF-α, MSCs secrete a large number of T-cell chemokines and upregulate anti-inflammatory cytokines such as TGF-β and IL-10, which in combination exert an inhibitory effect on T-cell proliferation, thereby inducing immunosuppression; in oral soft tissue inflammation, local application of MSCs can induce local IFN-γ levels to some extent, inhibiting the immune response and increasing the efficiency of tissue repair and regeneration. However, whether it is pro-inflammatory or anti-inflammatory effects, we believe that MSCs can, within a certain range, act as an immune regulator, sense the changes in the immune microenvironment, support the normal function of all kinds of immune cells in the environment, and drive the immune microenvironment towards regeneration and repair.

Inflammatory factors in the microenvironment also influence the direction and intensity of the immunomodulatory effects of MSCs (130). It has been shown that in response to inflammatory stimuli, DFSCs can highly express periosteal proteins (a bioactive molecule closely related to periodontal homeostasis), which can improve the immune microenvironment in the inflammatory region by regulating macrophages (131). In addition, the inflammatory microenvironment dominated by pro-inflammatory factors influences the osteogenic and odontogenic differentiation of MSCs. In a mouse periodontal model, inflammatory PDLSCs-EV promoted macrophage polarization towards the M1 phenotype and upregulated the level of local inflammation through miR143-3p-mediated modulation of the PI3K/AKT/NF-κB pathway (108), whereas inflammatory DPSCs-EV exhibited pro-inflammatory properties like PDLSCs-EV. In a 2019 study, researchers used lipopolysaccharide (LPS) from Porphyromonas gingivalis to mimic the inflammatory microenvironment of chronic periodontitis. Porphyromonas gingivalis (P. gingivalis) is one of the three well-documented major periodontal pathogens that play a key role in the pathogenesis of periodontitis by disrupting host immune homeostasis (132) and in this model, exosomes were extracted from periodontal ligament stem cells (PDLSCs) in the chronic inflammatory milieu and compared with normal PDLSC exosomes, and again found that microRNA-155-5p in inflammatory PDLSCs-EV increased SIRT1 expression in CD4^+^ T cells, thereby attenuating inflammatory response in chronic periodontitis (73). In the periodontal ecological niche, high levels of inflammation in the microenvironment reduce the osteogenic properties of PDLSCs, and these impaired PDLSCs may disrupt the microenvironment by exacerbating the host immune response, promoting aberrant angiogenesis and promoting osteoclast activity (133).

In fact, inflammatory factors often act in combination or synergistically to affect MSC function, for example, it has been found that GMSCs-EV generated by combined stimulation of TNF-α and IFN-α can promote the polarization of M2 macrophages by increasing the expression of CD73 and CD5L, which in turn enhances the anti-inflammatory function of the stem cells (134); IFN-γ can exert an inhibitory effect on osteogenesis of exogenous MSCs by enhancing TNF-α signaling and inhibition of the Runx2 pathway to exert an inhibitory effect on osteogenesis of exogenous MSCs (135), while TNF-α levels, when upregulated to a certain threshold, would have a combined effect with IFN-γ to promote apoptosis of endogenous and exogenous MSCs, which would adversely affect the periodontal regenerative environment.

### 4.2 Osteogenesis of MSCs based on the periodontal immune microenvironment

Firstly, MSCs in the organism have the function of osteogenic differentiation, which can be induced by BMP, TGF-β and other conditions to differentiate into osteoblasts and then produce bone-like structures or even form new bone. Second, endogenous and exogenous MSCs are able to secrete various osteogenic factors and bioactive substances to induce bone marrow stromal cells, connective tissue mesenchymal cells, etc., to transform into osteoblasts, and also to promote osteogenic differentiation of peripheral MSCs through paracrine effects. **Table 5** lists some representative studies on MSCs and MSCs-EV in periodontal bone regeneration in the last several years.

**Table 5:** Representative studies of MSCs and MSCs-EV in periodontal bone regeneration in the last several years.

Date	Cell type	Research model	Molecules Researched	Mechanism/channel	Effect	References
2024	PDLSCs	*in vitro*	EV-CircRNA_ 0000722	Up-regulating TRAF6 expression and activate downstream NF-κB and AKT signaling pathways	Promote osteoclast formation	Xie l et al. (136)
2023	PDLSCs	*In vivo* (mouse model) & *in vitro*	EV-miR-143-3p	miR-143-3p/PI3K/AKT/NF-κB pathway	Proinflammatory and promote M1 macrophage polarization	Wang Y et al. (108)
2023	DPSC	*In vivo* (rat model) & *in vitro*	DPSC-EV	IL-6/JAK2/STAT3 signaling pathways	Promote proliferation, migration and osteogenesis of PDLSCs *in vitro*, inhibit inflammation and promote tissue regeneration	Qiao X et al. (137)
2019	PDLSCs	*in vitro*	EV-MiR-155-5P	Th17/Treg/miR-155-5p/SIRT1 regulatory network	Down-regulation of periodontal inflammation	Zheng et al. (73)
2022	GMSCs	*in vitro*	GMSC-EV	NF-κB/Wnt5a	Downregulates inflammation and promotes regenerative capacity of PDLSC	Sun J et al. (138)
2021	GMSCs	*In vivo* (mouse model) & *in vitro*	EV-MiR-1260b	miR-1260b/Wnt5a/RANKL	Down-regulating inflammation and inhibiting bone resorption	Nakao et al. (45)
2023	BMSCs	*In vivo* (mouse model) & *in vitro*	EV-miR-1246	Down-regulating Th17/Treg	Anti-inflammatory effect, promote periodontal tissue regeneration	Xia Y et al. (66)
2020	BMSCs	*In vivo* (rat model) & *in vitro*	EV-miR-181b	PRKCD/AKT pathway	Promote M2 macrophage polarization, osteogenesis, anti-inflammatory	Liu W et al. (139)
2022	SHED	*in vitro*	SHED-EV	–	Promote periodontal tissue regeneration	Wang M et al. (140)

#### 4.2.1 MSCs maintain homeostasis in periodontal osteoimmunology

In bone tissue, a dynamic balance between osteoclastic bone resorption and osteoblastic bone formation is essential to maintain normal alveolar bone volume. The mechanism of communication between the immune system and this dynamic balance is called osteoimmunology. Viewed through the lens of osteoimmunology from the perspective of homeostatic medicine (141), periodontitis/periodontal bone loss occurs because the host immune system triggers a disturbance in the osteogenic/osteoclastic balance through several pathways, such as upregulation of inflammation and RANKL. Several studies have shown that the inflammatory cytokines IFN-γ, TNF-α and IL-1b secreted by Th cells not only upregulate local inflammation but also inhibit osteogenic differentiation; approximately 50% of T cells and 90% of B cells in gingival tissue from patients with periodontitis express RANKL (142), which is an important source of RANKL in the imbalance of bone immune homeostasis; numerous studies have shown that dendritic cells and osteoclasts co-operate to mediate bone destruction and remodeling under inflammatory conditions (143), and dendritic cells has even been suggested to intervene in the inflammation-induced osteogenic/osteoclastic balance as an osteoclast precursor; NK cells, in addition to non-specific killing of target cells, express RANKL (144) under inflammatory conditions in a manner that promotes osteoclast apoptosis and are involved in bone immunomodulation. MSCs and MSC-EV may intervene in these perturbing factors to maintain the balance between periodontal bone immune homeostasis and host inflammation. For example, using a rat apical periapical inflammation model, Xiao L et al. found that the interaction of BMSC with macrophages could regulate inflammatory bone loss in the SPHK1-S1PR1-RANKL axis (145); BMSC-EV, which has typical immunomodulatory, tissue repair, anti-inflammatory and antioxidant roles, modulated osteoclast production and osteogenic differentiation in periodontal inflammation, shifting the local microenvironment in a direction favorable to regeneration.

#### 4.2.2 MSCs-EV and ostoe-miRNA regulate the osteogenic potential of endogenous MSCs

MicroRNAs (miRNAs) are a class of endogenous non-coding small RNA molecules that are widely distributed in the body and regulate post-transcriptional target genes through complementary base pairing with mRNAs. miRNAs are considered to be an important component of stem cell biology, controlling the differentiation, immunomodulation and apoptosis of stem cells (odontogenic stem cells), as well as an important regulator of bone morphogenetic protein (BMP) (146). A 2022 study investigated whether SHED-derived exosomes could enhance the osteogenic capacity of hPDLCs (140). While the answer was positive, the researchers found a significant difference in miRNA content between SHED-derived exosomes and hPDLC-derived exosomes by analysis, suggesting that our miRNA has an important value in periodontal bone regeneration.

miRNAs can be carried by MSC-EVs and are being studied in many disciplines. For example, miR-1246 carried by hBMSC-EVs is a promising biomarker that has been used in many aspects of research, such as stem cell therapy, tumor immunity and elucidating the pathogenesis of certain diseases, and its use in tissue engineering is even considered as a potential key regulator of engineered DPSCs-derived exosomes (Ost-EVs) to promote periodontal tissue regeneration (147). In addition, in the body’s immune regulation, miRNAs may play a role in periodontal tissue regeneration by acting on various targets to regulate the signaling of key transcription factors (e.g. STATs) (148) and control the polarization and activity of cells such as macrophages. It has been shown that miR-1246 promotes M2 macrophage polarization and attenuates inflammation through the Wnt pathway, which in turn recruits MSCs to promote osteogenic differentiation.

ostoe-miRNAs refer to osteogenic differentiation-associated miRNAs, which are closely linked to the regulation of osteoblast differentiation and bone formation and accelerate bone healing through bone immunomodulation. For example, there is evidence that molecules such as miR-218 and miR374a can directly/indirectly promote osteogenesis of MSCs (ADSCs, PDLSCs) through the Wnt pathway (149, 150) and that down-regulation of their expression significantly inhibits the osteogenic differentiation of stem cells; several studies have shown that salivary expression of miR146a and miR155 is significantly increased in periodontitis patients (151, 152). They inhibit osteoclast genesis and stimulate activation of the NF-κB pathway. In addition, miR146a has been shown to be a predictive indicator of periodontitis in diabetic patients.

Studies on the involvement of miRNA-overexpressing MSC-EVs in immune regulation and bone metabolism have increased in recent years. Liu W et al. demonstrated that overexpressed EV-MiR-181b could promote hBMSC osteogenesis *in vitro* and bone healing *in vivo* by modulating the PRKCD/AKT pathway, promoting polarization of M2 phenotypic macrophages and inhibiting inflammatory responses (139). Nakao Y et al. found that miR-1260b overexpressed in GMSC-EV after inflammatory stimulation inhibited osteoclast activity by targeting the Wnt5a-mediated RANKL pathway (45) and promoted periodontal bone regeneration. Another study claimed that MiR-758-5p, which was upregulated in inflammatory DPSC-EVs, exerted a facilitating effect on the osteogenic and odontogenic differentiation of PDLSCs (153). Despite the promising therapeutic potential of MSCs-EV and its miRNA content, a number of challenges remain, e.g., the interaction of multiple miRNAs with their targets and the identification of the optimal miRNA therapeutic combinations are areas that require further research (154).

## 5 Research frontiers in the mechanisms of MSCs-regulated periodontal regeneration: complex multi-pathway networks and multiple perspectives

**Figure 2:**
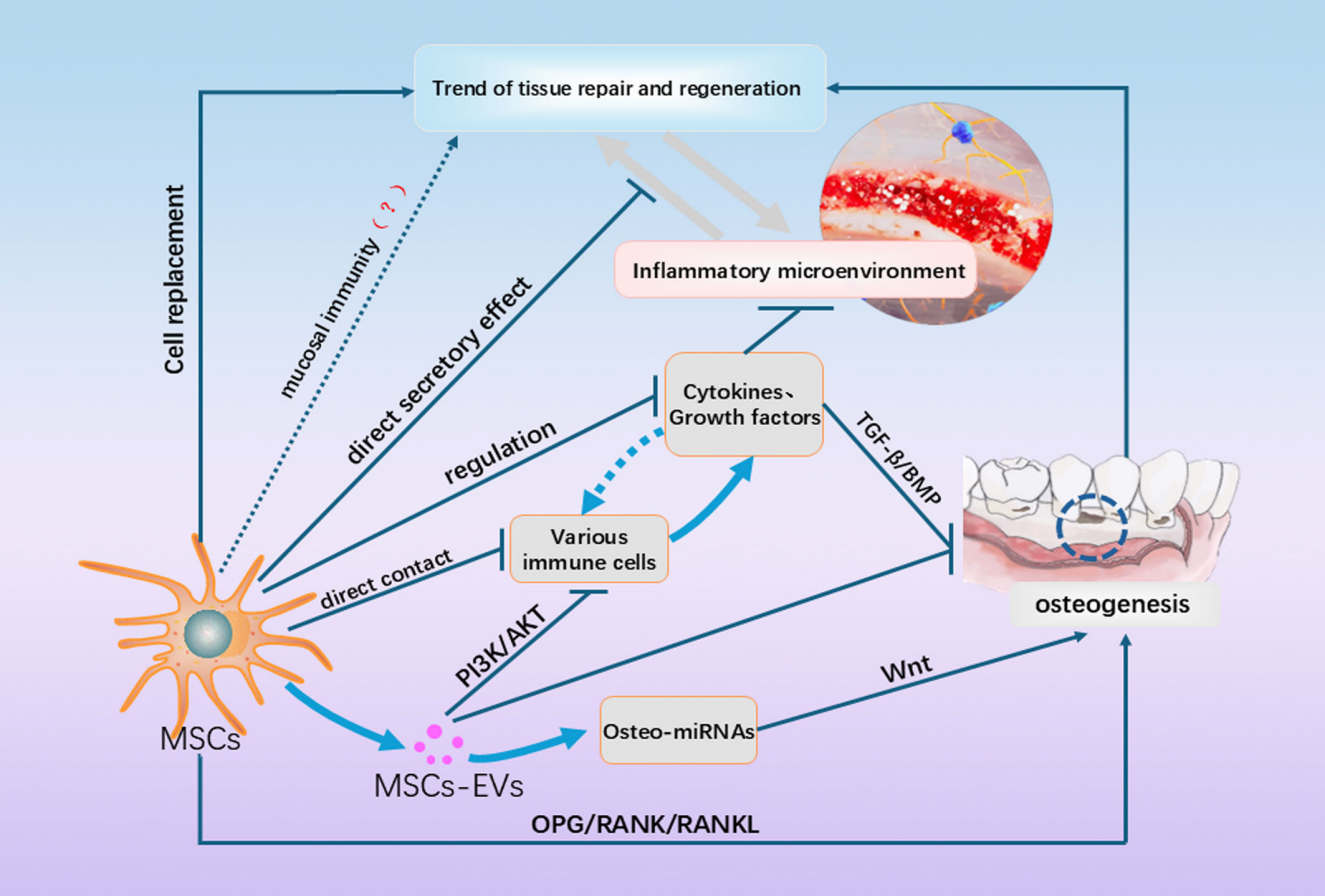
This figure shows a partial of the currently known and unknown mechanisms by which MSCs promote periodontal regeneration. In addition to cell replacement, direct effects on immune cells and the immune microenvironment, they also include paracrine secretion and influence on OB/OC balance, etc. In addition, the inflammatory and regenerative microenvironments could have shifted to each other under certain conditions, and the regenerative effects of MSCs on the microenvironment as influenced by the microenvironment are not static, and more of these mechanisms need to be explored in the future.

It is easy to see from the previous parts of the discussion that alveolar bone and periodontal tissue regeneration is a multifactorial synergistic process. It involves biomolecules and signaling pathways such as EV-miRNA, TGF-β/BMP, Wnt, PI3K/AKT, etc., which can act synergistically with each other and inevitably influence each other, resulting in a complex regulatory network (**Figure 2**). From controlling inflammation to maintaining the osteogenic/osteoblastic balance, the immunomodulatory effects of MSCs permeate the regulatory network of periodontal regeneration. As research continues to improve, more potential regulatory mechanisms remain to be discovered in the future. Below we will discuss a few research directions that have seen rapid growth in recent years.

### 5.1 The value of MSCs and epigenetic regulation in periodontal bone regeneration

Epigenetics refers to changes in gene expression during cell proliferation and division without altering the DNA sequence of the relevant genes, and its main molecular mechanisms include DNA methylation, histone modification, chromatin remodeling, non-coding RNAs, etc. In both heredity and disease development, the external environment can affect gene expression through epigenetics, resulting in biological phenotypic differences. A large body of evidence suggests that epigenetic mechanisms play an important role in the functional regulation of MSCs, which can influence the biological properties of MSCs and the regenerative potential of the participating bone-forming cells, and therefore are important for promoting periodontal bone regeneration and bone repair.

Histone acetylation is a post-translational histone modification that plays an important role in promoting osteogenic and dentinogenic differentiation of MSCs. In studies of periodontal tissue regeneration, histone deacetylase inhibitors (HDACi) have been shown to regulate osteogenesis and control inflammation by affecting epigenetics. It can promote MSC-mediated periodontal bone regeneration through mechanisms such as increasing the synthesis of osteogenesis-related proteins (e.g., osteoblasts, collagen-1α, and bone morphogenetic proteins) and inducing osteogenic differentiation of MSCs or osteogenic progenitor cells. In addition, both *in vitro* and animal studies have demonstrated the immunomodulatory ability of HDACi to inhibit T-cell activation and proliferation and to reduce the expression of pro-inflammatory factors. For example, it was shown that HDAC3 significantly reduced the expression of inflammatory mediators in gingival fibroblasts (GFs) from both healthy and diseased individuals in a model of periodontitis induced by TNF-α and Porphyromonas gingivalis (155). HDAC1 and HDAC3 can bind to NF-κB and promote the deacetylation process of NF-κB, thus inhibiting the inflammation mediated by NF-κB gene transcription and promoting periodontal tissue regeneration under inflammatory state.

Many studies have demonstrated that histone methylation plays an important role in regulating the dynamic balance of chromatin and the function of MSCs, and that histone methylation epigenetically regulates the differentiation of MSCs mainly by modifying gene transcripts. For example, in non-inflammatory environments, histone demethylases such as KDM6A, as well as certain miRNAs and lncRNAs, enhance the osteogenic capacity of PDLSC and promote osteogenesis. However, inflammation can inhibit osteogenic differentiation by upregulating DNA methylation of osteogenesis-related genes, modulating histone modifications, and causing alterations in noncoding RNAs (156); The histone demethylase KDM2A regulates the expression of the epidermal growth factor EREG and inhibits the osteogenic/odontogenic differentiation capacity of odontogenic MSCs and bone marrow-derived MSCs, thereby attenuating oral and maxillofacial bone regeneration.

Recent studies have shown that MSCs are also dependent on epigenetic modifications to differentiate into an osteoblastic lineage. Therefore, exploring the potential mechanisms of epigenetic regulation of MSC-driven differentiation may help us to enhance the activity and function of MSCs and provide new therapeutic targets for periodontal bone regeneration, which is a promising direction for future development (157). In addition, the important role of miRNAs in periodontal bone regeneration has already been mentioned in the previous section, and in fact, it was found that the expression levels of dozens of miRNAs changed significantly during the differentiation of MSCs into osteoblasts, including miR-30 family, let-7 family, miR-16, miR-21, miR-125, miR-155, etc. (158), which have strong or weak positive or negative regulatory effects on osteogenesis, and studies on these miRNAs in the past decade have been incessant, but at this stage, most of the studies on miRNAs are at the initial stage of cloning and screening specific miRNAs, and there is still a long way to go in our research on the functions, mechanisms, and applications of different miRNAs in different MSCs. There is still a long way to go for research on the function, mechanism and application of different miRNAs in different MSCs.

### 5.2 Oral mucosal immune homeostasis: a highly promising research area

Periodontal lesions are closely associated with oral mucosal immunity (159). Periodontitis has certain peculiarities compared to other diseases of bone immunity or bone loss. Since the oral cavity is a bacterial environment, immune dysregulation in the microenvironment of oral inflammation occurs on the basis of a disruption of the immune balance between bacteria and the mucosa, and the immune homeostasis of the oral mucosa influences the acute and chronic episodes of the disease and its severity in the early stages of inflammation or at the forefront of the disruption and disintegration of the soft tissues of the oral cavity (e.g. gingival tissues).Human commensal flora and mucosal immunity have been a hot topic of research in recent years, providing many new ideas for the treatment of a variety of mucosal-associated diseases. Regulatory T cell subpopulations exist in the mucosal epithelium and lamina propria (e.g. Th1, Th2, Th17, etc.) and their respective functions are in a state of equilibrium in mucosal immune homeostasis. When this balance is disturbed by certain factors at the right time, certain T cell populations within the mucosa become abnormal, resulting in mucosal inflammation and tissue destruction.

Interestingly, in contrast to periodontitis, where Th17 cells upregulate inflammation and promote osteoblasts, Th17 cells have been shown to regulate flora microecology and play an important role in maintaining tissue homeostasis and immune defense of the mucosal barrier. They also play a role in the prevention of infectious mucosal diseases (e.g. oral candidiasis) when triggered and induced by specific microbiomes (160, 161). The immune mechanisms at the oral mucosal barrier are less well studied, but it has been suggested that gingival Th17 cells accumulate independently of the commensal microbiota in healthy environments, and the trigger for Th17 cell accumulation in the gingiva is the sustained injury that occurs during mastication (162), and this localized tissue injury triggers the epithelial cells to produce IL-6, whereas the homeostatic Th17 cell accumulation in the oral cavity associated with mucosal health is dependent on this fraction of IL-6 production. accumulation is dependent on this IL-6. Homeostatic Th17 cells secrete IL-17 and mediate IL-17-dependent protective barrier responses such as antimicrobial defense. When periodontal inflammation spreads to the gingival tissue, the local flora associated with periodontitis acts on Th17 cells to expand in the gingiva due to the combined effect of IL-6 and IL-23 (163). At this point, the steady-state Th17 in the microenvironment is transformed into pathogenic Th17 cells, and the pathogenic Th17 leads to periodontal bone loss through immunopathological mechanisms such as excessive neutrophil recruitment and suppression of Treg cell function. This suggests to us that IL-23 in oral mucosal epithelial cells may play a pivotal role in the initiation of mucosal immune dysregulation in periodontitis.

In 2024, a study by Niki M. Moutsopoulos’ group (164) at the NIH demonstrated that epithelial cell-derived IL-23 is the initiator of inflammation in periodontitis, promotes the oral mucosal immune response to microbial components of the bacterial flora (e.g. flagellin, etc.), and serves as a mediator of inflammation in the subsequent development and regression of periodontitis. Using a mouse model of periodontitis, the researchers first demonstrated that IL-23A knockout mice were protected from periodontal bone destruction compared to wild-type mice, and then developed a mouse bone marrow transplantation (BMT) assay to determine that non-blood (epithelial) sources of IL-23 mediate the development of periodontitis. For IL-23, which is derived from hematopoietic and immune cells, existing studies appear to be more mature and MSCs can downregulate its mediated inflammatory response through multiple pathways such as expression of specific proteins and inhibition of Th17 activation. However, at this stage further studies are needed on the regulation of epithelial-derived IL-23 by MSCs with oral mucosal immunity.

**Figure 3:**
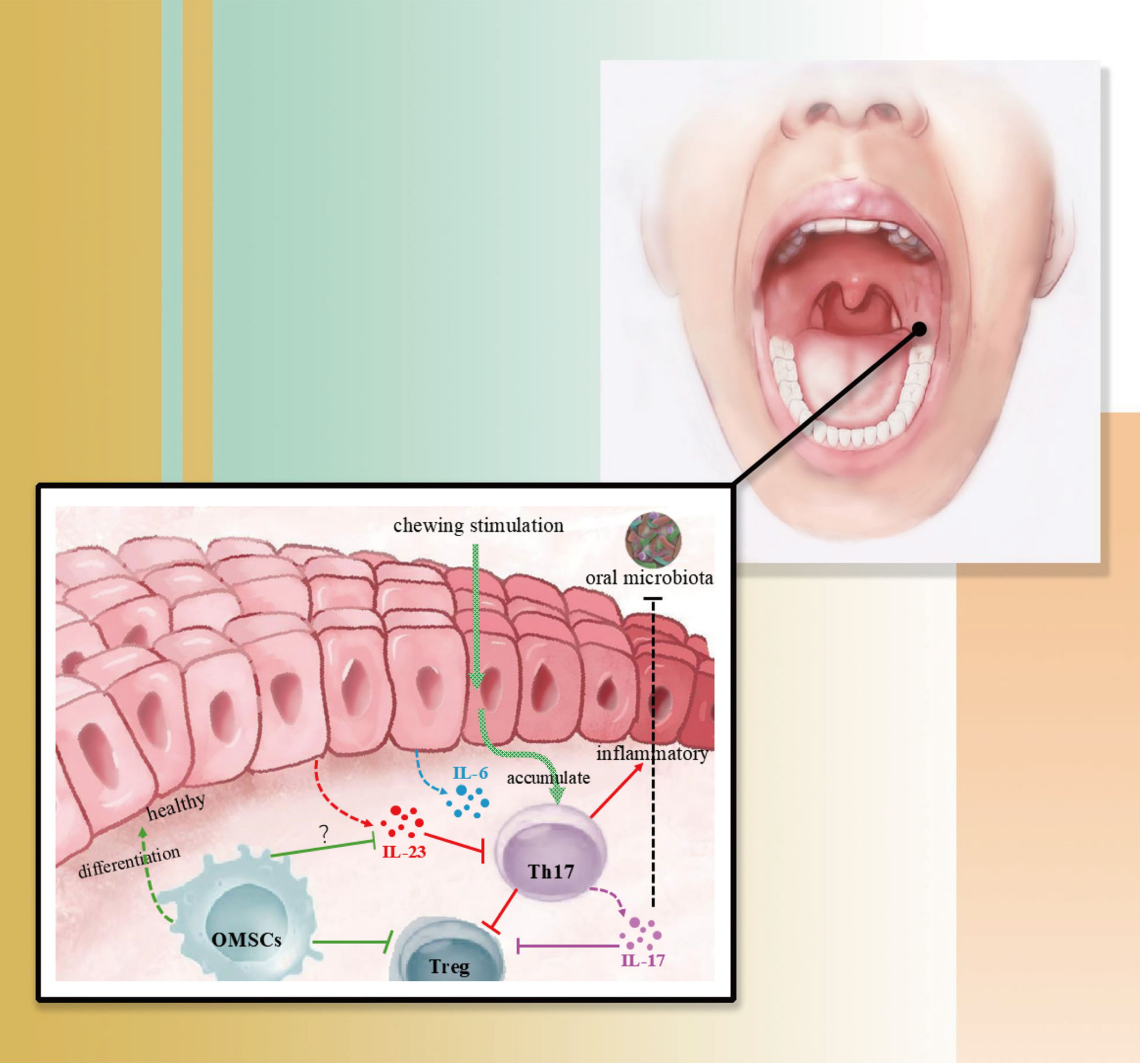
This figure shows a simple mechanism of immune homeostasis in the oral mucosa. In healthy gingiva, oral flora and mucosal immunity are in a state of equilibrium, where gingival irritation and damage during mastication causes Th17 to accumulate and triggers epithelial production of IL-6, a process that does not lead to inflammation; however, in the presence of epithelial-derived IL-23, the Th17-associated mucosal immune homeostasis is dysfunctional, leading to inflammation. The role of OMSCs located in the oral mucosa in the regulation of epithelial-derived IL-23 needs to be further investigated.

In recent years, the clinical concept of peri-implant tissue regeneration in implant restorations has subtly changed, with more and more senior clinicians recognizing the importance of soft tissue management (165), which is very different from the previous concept of focusing on bone augmentation, and believing that although bone augmentation is still important, soft tissue augmentation usually achieves the best results without the need for additional bone grafting. Therefore, it would have far-reaching clinical implications to combine the immunomodulatory effects of MSCs with oral mucosal immunity and to study their role in periodontitis as well as in periodontal soft tissue regeneration. In addition, oral mucosal stem cells (OMSCs) found in the oral mucosa have been shown to have a strong potential to differentiate into epithelial cells and maintain mucosal homeostasis through immunomodulation, are relatively easy to obtain and hold great promise for research (166, 167) (**Figure 3**).

### 5.3 Regenerative medicine therapies combining MSCs and small molecule drugs

Recent studies have found that combination therapy of MSCs with small molecules of natural and pharmaceutical origin significantly enhanced the efficiency and effectiveness of MSCs in bone regeneration, where polyphenols and flavonoids small molecules have shown preliminary promotive effects on periodontal bone regeneration. For example, Samiei et al. showed that the use of curcumin and calcitriol promoted the differentiation and mineralization of DPSCs (168). And curcumin stimulation increases ALP expression of mRNA in DPSC cells; flavonoids can protect periodontal tissues from oxidative stress damage by balancing free radicals and antioxidant properties (169).

Curcumin is a polyphenol known for its powerful anti-inflammatory, antioxidant and anticancer properties. As a small molecule compound of natural origin, polyphenols have been shown to exhibit potent antimicrobial effects against Porphyromonas gingivalis and Actinobacillus polymorpha, the two main bacteria of periodontitis (170), Polyphenols have also been shown to inhibit biofilm formation, e.g., curcumin and quercetin have been shown to disrupt biofilm structure and reduce bacterial viability (171), which is crucial in the treatment of periodontal disease. Polyphenols also modulate inflammatory pathways and reduce the expression of pro-inflammatory cytokines (e.g., tumor necrosis factor-α, IL-1β, and IL-6) (172). In addition, curcumin has been incorporated into topical drug delivery systems such as gels and nanoparticles in medical-industrial crossover experiments and tissue engineering to improve its stability and targeted delivery within periodontal pockets (173).

Flavonoids have been shown to promote periodontal tissue regeneration by enhancing osteogenic differentiation and increasing collagen secretion (174). In recent years, flavonoid-containing delivery systems have been widely explored in tissue engineering, such as thermosensitive hydrogels, to improve the retention and release of flavonoids at the site of periodontal lesions, thereby enhancing their therapeutic effects. Flavonoids can modulate inflammatory pathways, such as the Rank/NF-κB pathway, thereby decreasing the expression of inflammatory cytokines, such as IL-1β and TNF-α, in order to exert anti-inflammatory effects (175).

From the above, stem cell-based therapies have a bright future in combination with small molecule drugs, and not only that, but the combination of MSCs with therapies from other disciplines has shown great potential to improve the outcome of various diseases. These combinations both utilize and enhance the regenerative potential and immunomodulatory properties of MSCs and purposefully overcome the limitations of MSCs therapies. However, Amato et al. argue that it is important to consider the multifactorial etiology of periodontal disease in these processes, including systemic factors such as diabetes and oral microbiota imbalance. Addressing these underlying issues alongside regenerative treatments will improve the overall efficacy of periodontal inflammation control (176).

### 5.4 Tissue-resident macrophages: specific regulatory role for tissue regeneration and crosstalk with stem cells

Tissue-resident macrophages (tRMs) are specific immune cells that are found in specific tissues (e.g. peritoneal macrophages in the peritoneal cavity, TIM4^+^ self-renewing resident macrophages (SRRMs) in skeletal muscle) and are an integral part of the innate immune system (177). They play a crucial role in disease through immunosurveillance, promotion of post-injury tissue remodeling, and resistance to inflammation (178). For example, in inflammatory bowel disease (IBD), tRMs regulate immune dynamic homeostasis and epithelial regeneration. Their differentiation into pro- or anti-inflammatory phenotypes affects disease progression, highlighting their potential as therapeutic targets (179). In addition, tissue-resident macrophage population changes appear to be important during cancer progression. For example, in breast cancer, tRMs in the breast microenvironment can promote disease progression and metastasis. They interact with other immune cells and cancer cells to influence immunosuppressive or cytotoxic pathways (180).

As the understanding of macrophage phenotypes and subpopulations has increased, the crosstalk between macrophages and stem cells has been extensively studied. Similar to other types of macrophages, tRM can produce cytokines and growth factors that regulate stem cell activity and activate stem cells by modulating the inflammatory milieu, and recent studies have shown that in the mammary gland, resident macrophages regulate stem cell activity via the TNF-α-PI3K-CDK1/Cyclin B1 axis. This interaction is critical for maintaining breast stem cell activity and dynamic homeostasis5. In various tissues of the human body, different macrophage populations play a direct role in the generation of tissue-resident stem cells, including macrophages guiding stem cells into permanent niches (181) and generating transient niches to support proliferation during regeneration (182).

However, in recent years there has been a greater understanding of the role of macrophages in direct cell-cell interactions with stem cells, which may suggest a greater regulatory role within stem cell ecological niches. For example, Kim et al. demonstrated that macrophages play a direct role in stem cell ecotopia generation within the gastrointestinal tract (GIT), including direct sampling of stem cells for screening to ensure cell quality (183). Manneken JD et al. argued that organelle-organelle transfer between resident histiocytes, or MSCs, and macrophages appeared to be a mechanism that directly affects macrophage phenotypes, which is likewise a cell- cell interaction response and a mechanism used for systemic metabolic homeostasis (184).

In summary, studying the role of tRM within periodontal soft tissues in the regulation of periodontal health as well as periodontal inflammation, and elucidating the interactions between periodontal tRM and periodontal stem cells are extremely promising developments. Recent studies have shown that the generation of long-lived CD206+ tissue-resident macrophages, which colonize mesenchymal niche cells (MNCs) during early gut development, also requires appropriate microbiota colonization (183). Since the oral microenvironment is then heavily colonized by microbiota, we note that studies based on the ecological niche of periodontal stem cells may have a role in the interaction between them and tRM and between them and the oral microbiota. In these crosstalk, there are a large number of variables to be discussed, analyzed and therefore specific conditions need to be qualified, thus making these studies challenging.

## 6 Summary and outlook

The development of MSC-based cell therapy has revolutionary clinical implications compared to conventional periodontal therapy (185). MSCs therapy offers a viable new approach to eradicate the intrinsic cause of periodontal disease and promote the reconstruction of periodontal supportive tissues while alleviating the pain suffered by patients during conventional instrumental and surgical treatments. In preclinical studies, periodontal MSCs have shown a higher proliferation rate, while bone marrow MSCs have an unprecedented differentiation potential (186). In addition, PDLSCs have shown superior results in periodontal regeneration, particularly in enhancing periodontal ligament and bone regeneration compared to other MSCs (187). However, in the inflammatory environment, DFSC outperform PDLSC, suggesting their potential in specific pathological conditions (188). However, MSCs from other sources, such as adipose-derived stem cells, have also demonstrated their unique strengths in their respective directions, and therefore MSCs from different sources have their own strengths and value depending on the needs in terms of differentiation ability, regenerative outcomes, and clinical applications.

From the above sections, it is clear that MSCs and MSC-EV can play a role in various aspects of periodontal tissue regeneration through immunomodulation (including bone immunomodulation), and this has been demonstrated in both *in vivo* and *in vitro* models of periodontitis treatment. However, current studies of MSCs have not been able to quantitatively assess the impact of their immunomodulatory properties on the body and local environment. Marwa et al. discussed for the first time the extent to which cell source and cell mass can alter the cellular profile of immune cytokines and the extent to which such changes can affect immune cells (189) using bone marrow and adipose tissue derived stem cells as examples, providing a new direction for the future development of stem cell therapy. development by providing new directions.

Cell-free therapies achieved by paracrine secretion of MSC-EVs are a promising approach to periodontal regeneration, and their advantages over cell-based therapies include reduced risk of immune rejection, greater stability, and fewer ethical concerns. This makes them a viable option for a wide range of clinical applications in the field of periodontal regeneration (190). However, most studies on this treatment method are still at the preclinical research stage, awaiting further mechanistic studies and the next step of clinical trials. In 2019, Peng Hu et al. (191) with a large number of experiments, as well as the understanding of the pathological microenvironment at different stages of the disease, put forward the idea and suggestion that the isolation and extraction of MSCs-EVs need to be combined with the specific pathological microenvironment of the disease to be treated, and pointed out the scientific problems in the current “cell-free therapy” research direction through the hypothesis of MSCs-mexos. They believe that how to better combine with the microenvironmental conditions of the disease or injury site to stimulate or induce MSCs to “accurately” secrete MSCs-EVs related to the therapeutic disease and significantly enhance their secretion will be a key point to overcome for MSCs-EV to replace MSCs into clinical application. Periodontitis is precisely a disease that can go through acute and chronic phases, with multiple phases of inflammation, and is difficult to cure completely. As introduced in the previous section, there are a number of studies investigating the interaction of the periodontal inflammatory microenvironment on the properties of MSCs, and the results are not the same. Therefore, for the regeneration of periodontal tissues or the precise treatment of periodontitis, the study of the inflammatory immune microenvironment needs to open new horizons, which is of great importance for the evaluation of the cellular therapeutic value of periodontal disease and periodontal bone regeneration.

Despite the promise of MSCs and MSCs-EVs therapy to promote periodontal regeneration, it inevitably suffers from some of the common problems faced by stem cell therapy, such as lack of cell sources, low implantation and proliferation efficiency, low survival rate, and insufficient ability to homing to target tissues (192). Therefore, more research is needed to fully understand its potential and limitations. In addition, the use of stem cell therapy in periodontitis faces challenges of standardization, long-term evaluation, and ethical considerations. Patient selection criteria and treatment protocols are critical for successful implementation. The current lack of comprehensive clinical evidence and the need for responsible regulatory compliance highlight the importance of continued research and development in this field (36). Overcoming the challenges of production, scalability and therapeutic efficacy in clinical trials will overcome the limitations of current MSC and MSC-EV therapies. Spherical culture of MSCs has been shown to improve the therapeutic efficacy of the cells and their exosomes. This three-dimensional culture method improves the production rate and therapeutic properties of MSC exosomes, which is essential for effective cell therapy applications (37). In MSC-EV therapy, therapeutic efficacy can be greatly improved by tailoring to specific clinical indications and using biomaterials for efficient delivery and controlled release (38). Research into a new production method that would enable the industrialization and large-scale production of EVs is also a pressing issue at present.
